# Implementation of a heat-responsive care management initiative for Medicare advantage members residing across the United States

**DOI:** 10.1016/j.joclim.2026.100769

**Published:** 2026-10-28

**Authors:** Amanda L. Zaleski, Omar Soliman, Annie Laurie E. Hines, Matthew G. Anderson, Eric G. Hamilton, Amanda E. Cheng, Eric A. Simoni, Daniel B. Knecht, Paige G. Wickner, Sreekanth K. Chaguturu

**Affiliations:** a CVS Health®, 93 Worcester Street, Wellesley, MA, USA; b Haus Analytics, Inc, San Francisco, CA, USA; c EmblemHealth®, New York, NY, USA

**Keywords:** Climate adaptation, Heat exposure, Acute utilization, Health services research, Mitigation, Preparedness

## Abstract

**Introduction:**

Extreme heat events are increasing in frequency and intensity and disproportionally affect older adults and individuals with chronic conditions.

**Materials and Methods:**

Retrospective evaluation of a quality improvement initiative among care‑managed Medicare Advantage members of a U.S.-based payer randomly assigned to standard or enhanced outreach. Extreme heat forecasts were reviewed weekly to identify members in affected counties for outreach. Standard outreach involved routine nurse‑led telephonic care management. Enhanced outreach added brief heat‑specific education tailored to clinical risks. Outcomes included implementation measures and exploratory emergency department (ED) and inpatient admissions 30 days before and after heat‑triggered identification.

**Results:**

The cohort included 3,978 older adults (mean age: 75±9 y; 58% women) with high chronic disease burden. Standard care management outreach was delivered to 88.3% (3,514/3,978) of identified members, with no between-group differences (standard: 88.7% vs enhanced: 88.0%; *P*=0.53), demonstrating high fidelity. Engagement among outreached members was similar (standard: 86.5% vs enhanced: 86.7%; *P*=0.76). Heat-specific outreach was delivered to 17.3% of members assigned to enhanced outreach, with 79.5% engagement; limited crossover occurred in the standard group (2.9% outreached). ED visits and inpatient admissions declined modestly in both groups, with differences-in-difference estimates of -25 ED visits (95% confidence interval [CI]: -46 to 13; *P*=0.198) and -7 inpatient visits (95% CI: -44 to 46; *P*=0.84) per 1,000 members.

**Discussion and Conclusion:**

Heat‑responsive outreach was feasibly integrated into existing care management workflows while maintaining routine delivery and engagement. Future work should refine reach to evaluate clinical impact.

## Highlights

• Climate‑responsive outreach was integrated into routine care management workflows.
• Heat‑specific education was delivered without disrupting standard care management processes.
• No short‑term changes were observed in emergency or hospital use.
• Findings support scalable climate‑adaptive care approaches.

## 1 Introduction

Extreme heat events are increasing in frequency, intensity, and duration, creating urgent challenges for public health [1]. Since the 1990s, heat-related mortality has risen by 23%, contributing to >546,000 deaths annually [1], while excessive heat exposure causes heat-related illness and exacerbates cardiovascular, respiratory, renal, and mental health conditions [[2], [3], [4], [5], [6]]. These health effects translate into substantial economic and health system burden [1], including increased emergency department (ED) visits and hospitalizations [[7], [8], [9], [10], [11]]. In the United States (US), heat waves are associated with 10% and 7% higher incidence of heat-related ED visits and hospitalizations, respectively, compared with non-heat wave days [3], with costs estimated to exceed $1 billion each summer [12].

Health care systems are increasingly called on to play a proactive role in mitigating the health impacts of extreme weather events and strengthening preparedness to address climate‑related risks [13]. Public health agencies have implemented population-level interventions such as heat action plans, heat alert systems, early warning networks, and community-based education programs [[14], [15], [16], [17]]. These strategies can reduce mortality and improve public awareness of protective behaviors [15,[18], [19]], but are often designed for broad population coverage and can be challenging to personalize at scale. Scalable heat response systems require real-time identification of at-risk individuals and delivery of tailored, actionable guidance; however, many health systems lack the infrastructure to embed individualized outreach during rapidly evolving heat events [17].

Health insurers (often referred to as payers in the US) are uniquely positioned in the healthcare ecosystem to address these gaps through access to administrative claims data, predictive analytics, and established communication channels that enable timely identification and outreach to individuals at elevated risk. In the US, Medicare is the federal health insurance program for adults aged 65 years and older and certain younger individuals with qualifying disabilities. Medicare Advantage (MA) plans are privately administered health insurance plans that contract with the federal government to provide Medicare-covered benefits. In addition to insurance coverage, many MA organizations offer nurse-led care management services that provide health education, care coordination, and chronic disease support for members with complex health needs. These existing relationships and operational infrastructures may facilitate the delivery of personalized climate-responsive interventions at scale. Despite this potential, evidence on payer-led, individualized outreach during heat-related events is limited.

To address this, a quality improvement (QI) initiative was designed within a learning health system framework to deliver personalized outreach during extreme heat events for MA members with elevated clinical risk, leveraging existing nurse care management workflows. One-year post-implementation, this study aimed to evaluate the feasibility and implementation of a heat-responsive care management strategy and explore preliminary differences in 30-day ED and inpatient utilization between standard and enhanced outreach groups. It was hypothesized that enhanced, heat-specific outreach would be feasible to implement, with utilization outcomes evaluated as exploratory.

## 2 Methods

### 2.1 Pilot overview

This initiative was operationalized as a quality improvement pilot informed by implementation science principles. The goal was to evaluate whether climate-responsive outreach could be integrated into existing care management workflows and to establish baseline implementation benchmarks under real-world conditions.

MA members actively enrolled in an existing nurse-led care management program were identified using International Classification of Diseases, 10th Revision (ICD-10) codes for prespecified chronic conditions associated with impaired thermoregulation or elevated heat-related risk, including asthma, chronic obstructive pulmonary disease, hypertension, congestive heart failure, kidney disease, diabetes mellitus, and metabolic syndrome (Fig. 1). Care management services are routinely provided to eligible members and include telephonic health education, care coordination, chronic disease support, and navigation of healthcare services. Prior to the intervention window (i.e., 2024 heat season), 13,712 eligible care-managed members were randomized in a 1:1 ratio to receive either standard care management outreach or enhanced outreach that included additional heat-specific education. Randomization was performed at the individual level using simple random assignment via an automated algorithm within the QI study data environment. Each eligible member was assigned a random draw from a uniform (0,1) distribution, with values ≥0.50 assigned to the enhanced outreach group and values <0.50 assigned to the standard outreach group. No blocking or stratification was used. Members remained assigned to their original outreach group throughout the intervention period.

**Fig. 1:**
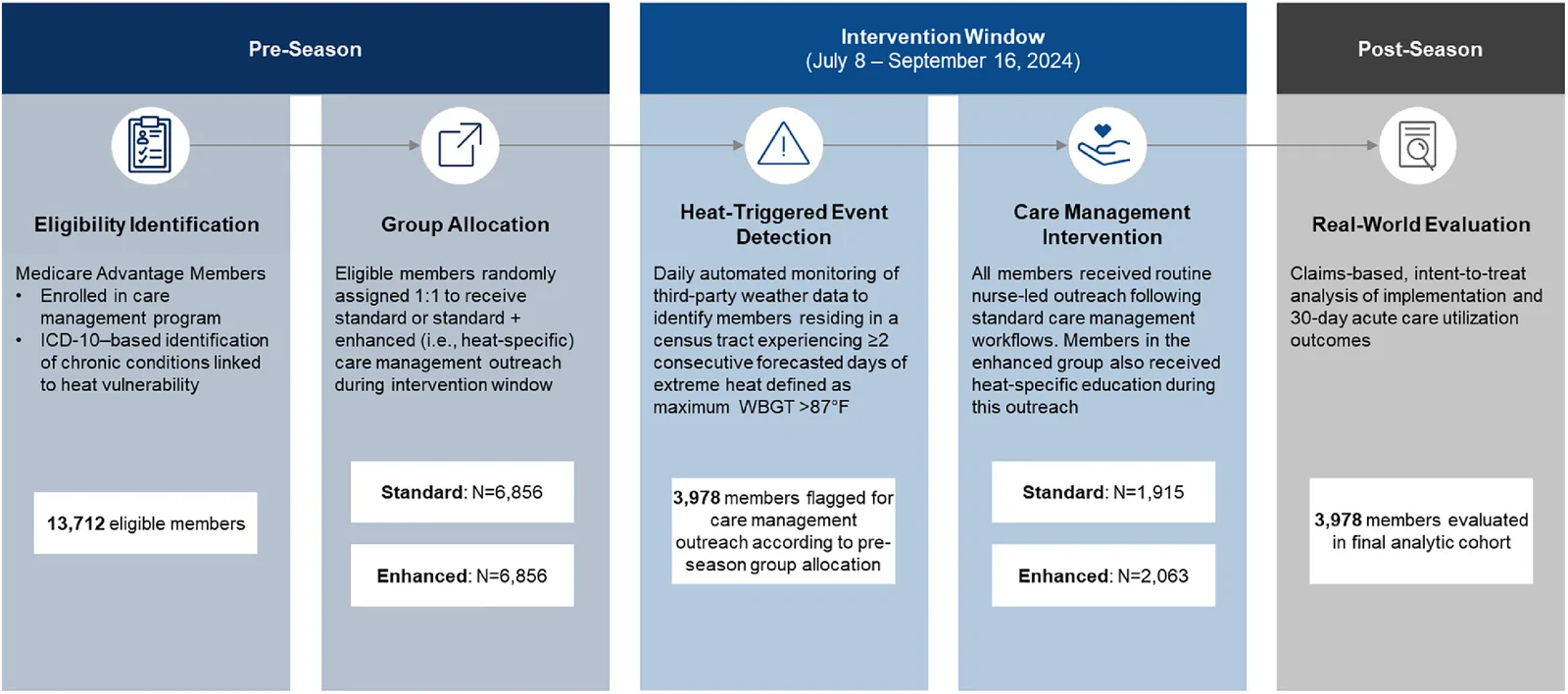
Conceptual Overview of a Quality‑Improvement Intervention to Identify Heat‑Vulnerable Members and Deliver Personalized Outreach During a Heat Wave Event Figure 1 Caption: Medicare Advantage members actively enrolled in care‑management programs were first identified using the 10th revision of the International Classification of Diseases (ICD-10) codes for chronic conditions associated with impaired thermoregulation or elevated heat‑related risk. Prior to the intervention window, all eligible members were randomized 1:1 to receive either routine care‑management outreach (standard) or an enhanced outreach protocol that included additional heat‑specific education. During the intervention period (July 8–September 16, 2024), ongoing monitoring of third‑party weather data identified census tracts with ≥2 consecutive days of forecasted extreme heat, defined as a maximum wet bulb globe temperature (WBGT) >87°F. Identification of a heat event triggered implementation of the quality‑improvement outreach workflow. Among 13,712 randomized members, 3,978 (29%) met heat‑triggered criteria and received outreach (standard: N = 1,915; enhanced: N = 2,063), forming the analytic cohort for implementation and 30‑day acute utilization analyses.

During the intervention window (July 8 ‒ September 16, 2024), forecasted weather conditions were monitored using third-party data supplied by a commercial weather intelligence vendor. Wet bulb globe temperature (WBGT), a composite measure of heat stress that incorporates temperature, humidity, solar radiation, and wind conditions, was used to identify forecasted extreme heat events [20]. Forecasted environmental conditions were geographically linked to members using residential census tract information. An extreme heat event was defined *a priori* as a WBGT >87°F on at least two consecutive days within the 10-day forecast period. This threshold was selected because it is associated with elevated risk of heat-related illness in the US [20]. Members residing in census tracts meeting these criteria were identified for outreach. During the intervention period, 3,978 members (29%) resided in locations meeting predefined extreme heat criteria and formed the analytic cohort.

Identification of a heat event triggered implementation of the QI protocol within existing care management workflows. Nurse care managers conducted standard telephonic outreach to all members as part of routine services, while members in the enhanced group received additional, personalized heat-specific education. Administrative claims data were deidentified, aggregated, and analyzed following an intent-to-treat approach to compare 30-day acute care utilization outcomes between members assigned to standard versus enhanced outreach who resided in locations experiencing extreme heat events.

### 2.2 Intervention description

#### 2.2.1 Standard care management

All nurse care managers hold an active, unrestricted RN license and participate in continuing education to maintain and expand clinical competencies and integrate emerging best practices. Care management provides coordinated support for members with complex health needs, focusing on chronic condition management, acute care episodes, and prevention of avoidable admissions. Outreach is primarily telephonic-based and delivered by nurse care managers, who provide personalized support tailored to each member’s health status and care plan. Standard care management outreach is designed to (1) serve as a trusted source of evidence-based health education; (2) support members in managing chronic and acute conditions; and (3) deliver timely, relevant, and actionable information to promote informed health decisions. Educational content is grounded in clinical guidelines and reviewed by internal medical experts. Behavioral and communication strategies, including motivational interviewing and cultural competence, are embedded within standard care management protocols.

#### 2.2.2 Enhanced care management

Members in the enhanced group received standard care management outreach plus heat-specific education during forecasted extreme heat events. Additional details regarding intervention personnel, telephonic delivery methods, outreach cadence, operational supports, documentation standards, and implementation workflows are summarized in the Supplement (Table A). Outreach education included timely, contextual, and relevant information about forecasted heat events and their potential health impacts. Education was tailored to each member’s clinical history, including evidence-based strategies to mitigate heat-related risks, such as hydration, limiting outdoor activity during peak heat hours, awareness of local cooling centers, considerations for medications that may impair thermoregulation, and management of heat-sensitive medical devices. Enhancements focused on operational workflow and documentation standards embedded within existing care management protocols. This low-intensity intervention aimed to increase awareness of heat-related health risks and encourage health-promoting behaviors during extreme weather events.

#### 2.2.3 Pilot implementation

The pilot was developed and launched over a condensed timeline. Organizational readiness (i.e., overall preparedness), competencies (i.e., people-level skills) and capabilities (i.e., system-level resources) were evaluated during the planning phase to inform implementation priorities. Two priorities emerged: (1) procurement and integration of a third-party weather data feed, and (2) development of standardized workflows and documentation for heat-specific outreach within existing care management protocols.

A third-party weather data vendor was procured in October 2023, providing the foundation for environmental monitoring. The vendor (unnamed due to operational nature of vendor agreement) was selected following an internal assessment of available weather intelligence solutions based on national geographic coverage, forecast availability, integration capabilities, availability of multiple environmental parameters to support potential future expansion to additional climate-related use cases (e.g., air quality), and operational suitability for member-level heat surveillance. Additional information regarding weather data sources, underlying models, and validation activities is available upon request. Organizational readiness activities, including partnership alignment, senior leadership approval, and assignment of a dedicated implementation specialist, were completed prior to the technical build. From April to June 2024, operational parameters, including weather thresholds, member eligibility logic, and architecture flow, were finalized. Back-end logic was updated and infrastructure for data flow was set up, followed by file testing in late June 2024.

Training and resource support were provided through a centralized landing page and email communication distributed to care managers in the weeks preceding launch. Resources included a job aid for heatwave outreach, communication guides, quick tips for hydration and heat safety, medication considerations, and condition-specific guidance. Links to evidence-based resources, cooling station locations, and emergency preparedness checklists were also included. To support implementation readiness, two optional live training sessions were offered on July 5 and July 7, 2024 (30 min each), with approximately 67% attendance. Training covered heat-related health risks, use of outreach materials, workflow expectations, documentation procedures, and delivery of heat-specific education. The pilot went live on July 8, 2024, following completion of system integration and readiness activities.

#### 2.2.4 Event-triggered workflow activation

Extreme heat monitoring was enabled through a third-party weather data feed integrated via an application programming interface (API) into a centralized cloud environment. Forecast data were visualized in the Power BI data visualization platform to identify members with heat-sensitive conditions in census tracts forecasted to experience ≥2 consecutive days of extreme heat in the 10-day weather outlook. Secure routing from the enterprise data warehouse to an existing nurse-led care management platform allowed timely, data-informed activation of heat-specific interventions within existing care management workflows.

#### 2.2.5 Nurse-led care management workflow and documentation

A centralized care management platform enabled streamlined member-facing operations. Upon inbound referral to the platform, care managers were notified via a task manager trigger alert and performed standardized workflows for initial member intake, including: 1) pre-call research, coordination of benefits, and medical chart review; 2) quality dashboard review to identify existing gaps in care; 3) thorough medication review, including medication reconciliation; 4) completion and documentation of standardized assessments; and 5) escalation and/or communication with the members’ provider(s). Members were prioritized based on nurse assessment of clinical complexity and perceived risk and contacted by their usual care manager within 1–2 days of identification.

### 2.3 Data sources and study setting

Forecasted and observed WBGT data were obtained via a third‑party weather data feed accessed through an API. Forecasted WBGT values were used prospectively for operational identification of extreme heat events and member targeting. Observed WBGT values, derived from recorded meteorological conditions, were queried retrospectively via the same API to characterize actual heat exposure during the intervention period and to support assessment of intervention fidelity.

Deidentified administrative and medical claims data for MA members of a large national health plan were retrospectively analyzed. Sociodemographic information collected at enrollment included member-reported sex, age, race, and ethnicity. Member-level data were linked to plan benefit design and residential location characteristics, including ZIP code-level socioeconomic indicators. Medical claims captured health care encounters, including service dates, sites of care, procedure codes, and diagnosis codes. Data also included aggregated member-level chronic disease burden and predictive risk scores. Operational warehouse data characterized implementation outcomes, consistent with Standards for Quality Improvement Reporting Excellence (SQUIRE 2.0) reporting standards [21].

### 2.4 Ethics approval

This evaluation was reviewed and classified as exempt [45 CFR 46.104(d)(4)] by the Sterling Institutional Review Board (#11401). The heat-responsive outreach initiative was conducted as a quality improvement activity within routine healthcare operations, and the retrospective evaluation used aggregated, de-identified administrative and claims data. No compensation was provided.

### 2.5 Statistical analysis

Implementation outcomes were analyzed using a mixed-methods approach following Proctor’s implementation research framework [21], with emphasis on reach, fidelity, engagement, and feasibility. Reach was defined as the proportion of eligible care-managed members identified during extreme heat events. Fidelity was defined as the proportion of eligible members receiving at least one standard care management contact attempt. Engagement was defined as a successful two-way interaction with a nurse care manager following outreach. Feasibility was assessed through a multicomponent evaluation of the intervention's operational implementation, including weather-data integration, trigger logic performance, workflow activation, timeliness of outreach, and scalability indicators. Implementation outcomes were reported to characterize real-world feasibility and delivery within routine care management workflows. Observed rates were intended to provide context regarding operational implementation and not performance thresholds. Operational definitions for implementation outcomes are further described in Table B in the Supplement.

Retrospective analysis of deidentified and aggregated medical claims data compared utilization outcomes, and percent difference in ED visit and inpatient admission rates, between members in the enhanced and standard (i.e., comparison) outreach groups following an intent-to-treat design. For members with multiple outreach events within a 30-day period (N = 213), only the first outreach was included to avoid overlapping observation windows and double counting of ED visits, assuming the initial outreach to have the greatest impact.

The pre-intervention period was defined as 30 days prior to heat-triggered member identification and the post-intervention period was defined as 30 days post-identification. Baseline characteristics were compared between groups using two-tailed, unpaired *t* tests for continuous variables and chi-squared tests for categorical variables. Yates' continuity correction was applied, as appropriate, for categorical comparisons. Relative changes in 30-day ED visits and inpatient admissions were calculated separately for the enhanced and standard outreach groups. To assess the incremental effect of enhanced outreach relative to standard outreach, a difference-in-differences framework compared changes from the pre- to post-intervention period between groups. Negative values indicate greater reductions in the enhanced versus standard group. A negative binomial regression model estimated intervention‑associated differences in visit counts. The model included terms for treatment group, time period (pre vs. post), and a treatment-by-time interaction, with the interaction term estimating the differential change in utilization associated with enhanced outreach relative to standard outreach. Observation windows were equal across members; therefore, no offset term was included. Model coefficients were estimated on the log scale and subsequently back-transformed to calculate absolute differences in utilization per 1,000 members using the pre-period utilization rate in the standard outreach group as the reference. Corresponding 95% confidence intervals (CIs) were derived by applying the same transformation to the lower and upper bounds of the interaction term's Cl. This approach was chosen due to overdispersion in ED and inpatient utilization data, with most members having zero visits and a minority exhibiting recurrent events. Effect estimates are reported with corresponding 95% CIs and two-sided *P* values. A *P* value of ≤0.05 was considered significant.

## 3 Results

### 3.1 Cohort characteristics

Table 1 details baseline characteristics of the standard and enhanced outreach groups. On average, the total included sample population (Total: N = 3,978; standard: n = 1,915; enhanced: n = 2,063) consisted of older adults (mean age ± standard deviation: 75±9 y; 58% women) with a high burden of chronic disease. A slightly higher prevalence of diabetes mellitus was observed among members in the standard (63%) than enhanced (59%; *P*=0.02). There were no between-group differences in remaining baseline characteristics, indicating successful covariate balance.

**Table 1:** Baseline sociodemographic and clinical characteristics of standard and enhanced groups.

**Characteristic**	**Standard (N** **=** **1915)**	**Enhanced (N** **=** **2063)**	***P* Value**
Age, mean (SD), y	76 (9)	75 (9)	0.31
Sex^a^, no. (%)			
Female	1,107 (57.8%)	1,198 (58.1%)	0.87
Male	808 (42.2%)	865 (41.9%)	0.87
Race and Ethnicity, no. (%)			
American Indian / Alaskan Native	NR (<1%)^b^	NR (<1%)^b^	0.26^c^
Asian	NR (<1%)^b^	NR (<1%)^b^	0.88^c^
Black or African American	448 (23%)	445 (22%)	0.17
White	1,180 (62%)	1,291 (63%)	0.53
Unknown (Missing)	243 (13%)	278 (13%)	0.46
Hispanic / Latino	34 (2%)	36 (2%)	0.94
US region, no. (%)			
South	1,071 (56%)	1,144 (55%)	0.76
Midwest	502 (26%)	568 (28%)	0.35
Northeast	273 (14%)	272 (13%)	0.33
West	69 (4%)	79 (4%)	0.71
Chronic condition, no. (%)			
Hypertension	1,840 (96%)	1,974 (96%)	0.53
Diabetes mellitus	1,199 (63%)	1,217 (59%)	0.02
Congestive heart failure	1,161 (61%)	1,242 (60%)	0.79
Kidney disease	1,107 (58%)	1,175 (57%)	0.61
Chronic obstructive pulmonary disease	892 (47%)	953 (46%)	0.81
Asthma	356 (19%)	400 (19%)	0.55
Metabolic syndrome	111 (6%)	136 (7%)	0.30
Acute events (per 1000 members), no. (SD)			
ED visits	117 (398)	139 (477)	0.11
Inpatient admissions	180 (466)	192 (462)	0.39

a Self-reported during plan enrollment.

b Cell size counts <10 were suppressed to protect confidentiality.

c Yates’ continuity correction was applied for categories with small cell sizes Abbreviations: ED, emergency department; SD, standard deviation; NR, not reported; US, United States.

### 3.2 Heat event characteristics

The mean forecasted WBGT during the intervention period was 89.3°F (range: 87.0 –100.3°F). Event-triggered workflows were activated for forecasted heat waves during weeks 1 through 9 of the 10-week intervention window (i.e., July 8 ‒ September 16, 2024). These forecasts met predefined operational criteria for high-risk heat exposure, defined as ≥2 consecutive days exceeding the WBGT threshold of 87°F. Retrospective analysis of observed WBGT values closely aligned with forecasts, with a mean of 91.6°F (range: 87.0 – 100.6°F), supporting the validity of the forecast-based exposure classification.

### 3.3 Implementation outcomes

Foundational implementation processes functioned as intended, supporting feasibility the heat-responsive workflow, including reliable trigger logic, accurate eligibility identification, consistent operational cadence, and timely workflow activation, enabling evaluation of downstream identification, outreach, and engagement outcomes (Table B in the Supplement).

Among 13,712 care-managed members randomized prior to the heat season, 3,978 (29%) were identified for outreach via prespecified heat-related trigger events during the intervention window and included in the analytic cohort. Standard nurse-led care management outreach was delivered reliably to 88.3% (3,514/3,978) of members identified during a heat-related event, with no difference between groups (standard: 88.7% [1,698/1,915], enhanced: 88.0% [1,816/2,063]; *P*=0.53), demonstrating high fidelity to routine care management services (Fig. 2). Engagement among outreached members was similarly high and equivalent between groups (standard: 86.5% [1,469/1,698], enhanced: 86.7% [1,574/1,816]; *P*=0.76; Fig. 2).

**Fig. 2:**
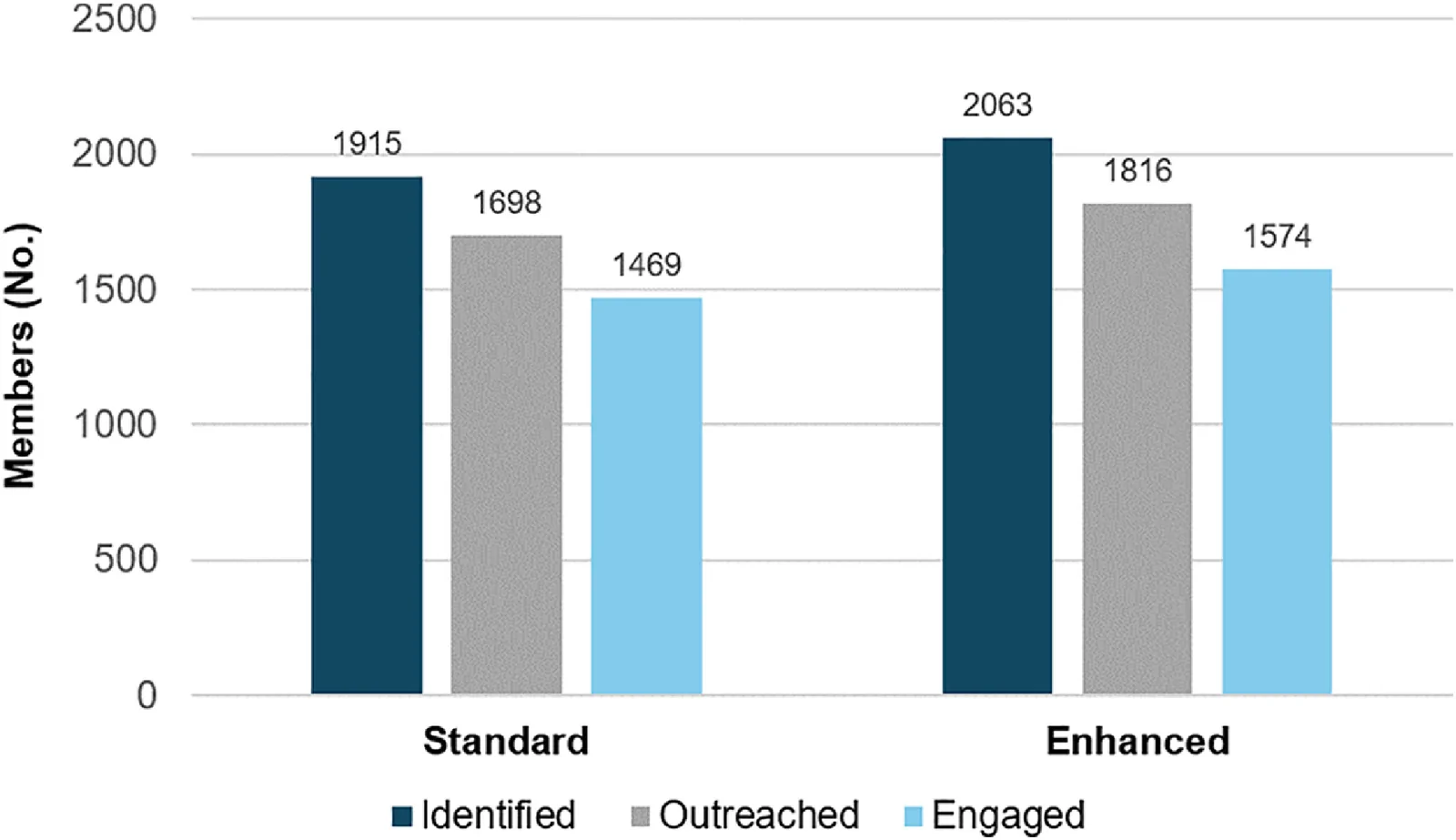
Fidelity to Routine Care-Management Services by Assignment Group (Standard versus Enhanced) Figure 2 Caption: Bars represent the number of members identified through prespecified heat‑related trigger events and the proportion subsequently outreached (of identified) and engaged (of outreached) for routine nurse‑led care‑management services. There were no between‑group differences in the delivery of routine, standard care‑management workflows.

Among members assigned to receive enhanced outreach, 17.3% (356/2,063) received heat-specific outreach with 79.5% (283/356) engagement. Limited crossover occurred, with 2.9% (56/1,915) of members assigned to receive standard outreach receiving heat-specific, enhanced outreach at nurse discretion; engagement among these crossover members was 96.4% (54/56), though absolute numbers were limited.

### 3.4 Healthcare utilization outcomes

Acute care utilization remained stable during the 30 days after heat-triggered identification compared with the 30 days before. Observed changes in ED visits were minimal in the standard outreach group (117 to 116 per 1,000 members) and declined in the enhanced outreach group (139 to 113 per 1,000 members). Inpatient admissions decreased in both groups, from 180 to 105 per 1,000 members in the standard group and from 192 to 110 per 1,000 members in the enhanced group. Difference-in-difference estimates were −25 ED visits (95% CI: −46 to 13*; P*=0.198) and −7 inpatient admissions (95% CI: −44 to 46*; P*=0.84) per 1,000 members, indicating no meaningful between-group differences.

## 4 Discussion

### 4.1 Principal findings

This retrospective evaluation of a payer-led QI initiative examined whether climate-responsive outreach could be integrated into existing care management infrastructure to support MA members during extreme heat events. As climate-related health risks continue to increase globally, healthcare organizations are seeking practical approaches to identify and support individuals at elevated risk during extreme weather events. This initiative leveraged existing clinical workflows, personnel, and technology systems to operationalize a climate-responsive care model within a learning health system, at scale.

Consistent with the study hypothesis, nurse-led delivery of heat-specific education to at-risk older adults during extreme heat events was feasible to implement with high fidelity to standard care management delivery and engagement. The intervention successfully integrated environmental surveillance, claims-based risk stratification, workflow activation, and telephonic outreach into existing care management operations. Collectively, these findings provide an early benchmark for implementation of climate-responsive interventions within routine healthcare delivery, including expected identification yield, workflow integration, implementation feasibility, and downstream member engagement under real-world conditions.

As an exploratory outcome, acute care utilization was evaluated following implementation. Overall, 30-day acute care utilization declined during the post-intervention period in both groups, with directionally larger reductions observed among members assigned to enhanced outreach.

### 4.2 Interpretation of implementation findings

Heat-related illnesses and mortality are largely preventable through planning, education, and behavior change. Population-level strategies such as public warnings, heat-health warning systems, and community outreach have demonstrated reductions in morbidity and mortality [15,18,[22], [23], [24]]. However, these approaches often lack individualized targeting and integration into clinical workflows. Clinic-based initiatives, including resilience toolkits and real-time alerts, have demonstrated feasibility, yet most models are provider-centric and are not designed to scale across geographically diverse populations [[25], [26], [27]].

The objective of the present initiative was to evaluate whether climate-responsive outreach could be operationalized within existing care management infrastructure and to characterize implementation performance under real-world conditions. As a learning health system pilot, the initiative sought to establish baseline implementation benchmarks related to member identification, workflow activation, outreach delivery, engagement, and operational feasibility. Findings from this pilot demonstrated that payer-enabled climate-responsive outreach can be incorporated into existing care management infrastructure and routine clinical workflows without disrupting standard care delivery. Claims-based risk stratification and real-time weather integration enabled timely, actionable guidance for at-risk members during forecasted extreme heat events.

A notable implementation finding was the difference between intervention assignment and successful delivery of heat-specific education. Among the 2,063 members assigned to enhanced outreach, 356 ultimately received the enhanced intervention. The volume of members qualifying for outreach was determined by the timing, geographic distribution, and severity of forecasted extreme heat events and therefore could not be fully anticipated in advance. Successful identification of at-risk members did not always translate into successful intervention delivery, highlighting intervention reach as an important implementation outcome and an area for future refinement. Future work should evaluate strategies to improve intervention reach and scalability while maintaining fidelity to routine care management operations.

Collectively, these findings extend the existing evidence base by demonstrating that a payer-enabled approach can help bridge gaps between broad public health messaging and individualized care. The study also provides practical insight into integrating climate-responsive interventions within routine healthcare delivery and highlights intervention reach as an important consideration for future scale-up efforts.

### 4.3 Practical implications for climate-responsive care

The implementation experience offers practical insights for healthcare organizations seeking to develop climate-responsive service models. Core components of the initiative, including environmental surveillance, risk stratification, workflow activation, targeted education, and integration into existing clinical operations, may be adaptable across diverse healthcare settings regardless of the underlying financing structure or care delivery model.

Although implemented within a MA population in the US, the operational lessons from this initiative may inform climate-responsive healthcare strategies in other countries facing increasing risks from extreme heat events. More broadly, the study demonstrates how existing healthcare infrastructure can be leveraged to identify vulnerable populations, deploy targeted interventions, and support climate adaptation without requiring creation of entirely new care delivery systems. The findings also highlight the value of learning health system approaches for establishing implementation benchmarks, identifying operational bottlenecks, and informing iterative refinement of climate-responsive care models.

### 4.4 Interpretation of healthcare utilization findings

Among members identified during extreme heat events, acute care utilization declined during the 30-day post-intervention period in both groups, with directionally greater reductions observed among members assigned to enhanced outreach. However, these differences were modest and did not reach statistical significance. While these findings may suggest the possibility of incremental benefit, they should be interpreted as exploratory and hypothesis-generating. Furthermore, acute care utilization does not inherently distinguish between potentially preventable utilization and appropriate healthcare-seeking behavior, underscoring the need for cautious interpretation of these exploratory outcomes.

High baseline engagement, limited intervention reach, and a short observation window may have attenuated detectable differences in this early-phase initiative. Additionally, although all members met predefined extreme heat conditions, observed utilization patterns also may have been influenced by broader environmental, seasonal, and healthcare utilization trends occurring during the study period. This evaluation was designed primarily to assess implementation feasibility rather than isolate the independent effects of changing heat exposure; therefore, the contribution of these factors cannot be determined.

### 4.5 Limitations

This study has several limitations. First, the sample was limited to members from a single national health plan, which may limit generalizability. Second, real-world capacity constraints limited outreach to 356 of 2,063 members assigned to the enhanced outreach group, likely attenuating observable differences in outcomes under an intent-to-treat design. Nevertheless, the observed reach provides valuable insight into the opportunities and challenges of integrating climate-responsive interventions into existing healthcare infrastructure under real-world conditions. Future studies may consider per-protocol or as-treated analyses, or strategies to increase outreach rates, to better understand potential impact. Third, utilization outcomes were inferred based on all-cause inpatient and ED utilization without direct assessment of heat-related clinical events. Mortality outcomes were not available for analysis and the study was not designed to evaluate heat-related mortality, which may be an important outcome for future investigations. Fourth, as a retrospective evaluation of a QI initiative, unmeasured confounding and secular trends in utilization may have influenced results despite randomization and the difference-in-differences design. Exposure assessment was based on geographically assigned environmental conditions rather than direct measurement of individual heat exposure. Individual-level factors including indoor temperatures, air conditioning access, mobility patterns, and time spent outdoors were unavailable and may have contributed to exposure misclassification. Changes in healthcare utilization also may have been influenced by factors beyond the intervention itself, including seasonal variation and temporal differences in environmental conditions. Although all members included in the analytic cohort met predefined extreme heat exposure criteria, the study was not designed to evaluate the independent impact of variation in extreme heat event frequency or intensity on utilization outcomes. Additionally, the negative binomial difference-in-differences model was specified at the observation level and did not explicitly account for within-person correlation between pre- and post-intervention utilization measurements. Given the null findings and wide confidence intervals, accounting for within-person correlation would be unlikely to alter the overall interpretation of the results. Fifth, voluntary participation introduces potential selection bias, although both groups were engaged in existing care management programs. Sixth, administrative claims data are subject to coding inaccuracies and missing data; however, both groups were derived from the same payer with consistent oversight mechanisms. Seventh, limited cross-over occurred, with 56 members (2.9%) assigned to standard outreach receiving heat-specific education, which may have attenuated between-group differences. Finally, extreme heat exposure was defined using WBGT thresholds and different cutoffs may yield different results, particularly in regions where baseline conditions differ from those observed in this study.

Despite these limitations, this study is among the first to assess large-scale, payer-led interventions designed to mitigate heat-related health risks through individualized outreach. The evaluation leveraged a real-world, claims-based design integrated with existing care management infrastructure, enabling implementation at scale across a geographically diverse MA population. Integration of member-level data with third-party weather data through interoperable systems enhanced targeting precision and operational feasibility. Access to administrative claims data enabled real-world quantification of outcomes, providing an early benchmark to inform future program development.

### 4.6 Future research

Implementation learnings and preliminary findings from this pilot have important implications for future research and practice. Larger, prospectively designed studies with sufficient power are needed to more precisely estimate the effects of heat-focused outreach; examine dose–response relationships (e.g., number and timing of contacts); and evaluate heterogeneity of treatment effects across subgroups defined by age, comorbidity burden, social risk factors, and baseline utilization.

Future studies should continue to evaluate both implementation and effectiveness outcomes of climate-responsive interventions across diverse healthcare settings. Adapting climate-responsive workflows to local operational contexts will be important for strengthening healthcare resilience to increasing climate-related risks. Future evaluations should also assess implementation strategies that improve intervention reach and scalability while maintaining fidelity within routine healthcare operations, including the use of operational dashboards and workflow analytics to better understand outreach prioritization, care manager staffing ratios, intervention dose, and time required for program delivery. Incorporating digital modalities, caregiver engagement, and automated workflow activation may further improve reach and engagement beyond telephonic outreach.

Expanding this model to other extreme weather events may inform broader climate-resilient care management strategies. Further evaluations should continue to evaluate outcomes across the quintuple aim of healthcare, including clinical outcomes, patient experience, cost, health equity, and care team well-being. Ongoing collection of mixed-methods feedback from members and care managers will inform continued program refinement and help shape future climate-responsive care models. Preliminary qualitative feedback suggest care managers highly value the program as a unique opportunity to deliver holistic support during moments that matter, while members value personalized and contextual guidance, including practical recommendations such as pet safety, heat preparedness, and awareness of local cooling resources.

## 5 Conclusion

This US-based, payer-led QI initiative demonstrates the feasibility of integrating climate-responsive outreach into routine care management workflows for MA members at elevated clinical risk during extreme heat events. No between-group differences in acute care utilization were observed, highlighting the need for further evaluation focused on optimizing reach and delivery. Nevertheless, these findings provide actionable implementation insights to inform scalable, climate-resilient care models.

## Disclaimer

The views expressed in this publication are those of the authors.

## Funding support

This study was funded by CVS Health® Corporation.

## Data sharing statement

The data sets generated during and/or analyzed in this evaluation are not publicly available due to commercial data use agreements.

## CRediT authorship contribution statement

**Amanda L. Zaleski:** Writing – original draft, Visualization, Software, Resources, Project administration, Methodology, Investigation, Conceptualization. **Omar Soliman:** Writing – review & editing, Methodology, Investigation, Formal analysis, Conceptualization. **Annie Laurie E. Hines:** Writing – review & editing, Project administration, Methodology, Conceptualization. **Matthew G. Anderson:** Supervision, Methodology, Investigation, Formal analysis, Data curation, Conceptualization. **Eric G. Hamilton:** Validation, Supervision, Software, Resources, Project administration, Methodology, Investigation, Funding acquisition, Formal analysis, Data curation, Conceptualization. **Amanda E. Cheng:** Project administration, Methodology, Investigation, Data curation, Conceptualization. **Eric A. Simoni:** Project administration, Investigation, Conceptualization. **Daniel B. Knecht:** Resources, Project administration, Investigation, Funding acquisition, Conceptualization. **Paige G. Wickner:** Supervision, Resources, Project administration, Methodology, Investigation, Funding acquisition, Data curation, Conceptualization. **Sreekanth K. Chaguturu:** Supervision, Investigation, Funding acquisition, Conceptualization.

## Declaration of competing interest

The authors declare the following financial interests/personal relationships which may be considered as potential competing interests: Amanda Zaleski reports financial support was provided by CVS Health Corp. Amanda Zaleski reports a relationship with CVS Health Corp that includes: employment and equity or stocks. Omar Soliman, Matthew Anderson, Eric Hamilton, Amanda Cheng, Paige Wickner, and Sreekanth Chaguturu reports a relationship with CVS Health Corp that includes: equity or stocks. Paige Wickner is also employed by Brigham and Women’s Hospital. Sreekanth Chaguturu is also employed by Massachusetts General Hospital. Daniel Knecht and Eric Simoni are former employees of CVS Health and are current employees at EmblemHealth®. Annie Laurie Hines was a former employee of CVS Health and is now a current employee at Haus Analytics, Inc. If there are other authors, they declare that they have no known competing financial interests or personal relationships that could have appeared to influence the work reported in this paper.

## Footnotes

Supplementary material associated with this article can be found, in the online version, at doi:10.1016/j.joclim.2026.100769.
