# Supplementary material for: Implementation of a heat-responsive care management initiative for Medicare advantage members residing across the United States
Source: J Clim Chang Health. 2026 Oct 28;32:100769. doi: 10.1016/j.joclim.2026.100769 (PMC13742314; doi:10.1016/j.joclim.2026.100769)
Supplement: Supplementary file 1 [file mmc1.docx]

**eSupplement**

**Elements (2)**

**eTable 1. Core Components of a Heat-Responsive Care Management Outreach Intervention**

**eTable 2. Implementation Outcomes of a Heat-Responsive Care Management Quality Improvement Initiative**

**eTable 1. Core Components of a Heat-Responsive Care Management Outreach Intervention**

| **Intervention Component** | **Description** |
| --- | --- |
| Intervention purpose | Proactive outreach to mitigate heat-related health risks among high-risk Medicare Advantage members during extreme heat events |
| Target population | Medicare Advantage members enrolled in care management with prespecified chronic conditions associated with elevated heat-related risk, identified via ICD-10 codes |
| Triggering mechanism | Forecasted extreme heat events identified using third-party weather data (wet bulb globe temperature >87◦ F on ≥2 consecutive days) |
| Risk stratification method | Claims-based identification of prespecified chronic conditions using administrative data. Members identified for outreach were prioritized by nurse care managers based on assessment of clinical complexity and perceived risk within routine care management workflows. |
| Delivery personnel | Nurse care managers operating within existing care management programs |
| Delivery modality | Telephonic outreach conducted during forecasted extreme heat events |
| Core intervention content | Heat-specific education tailored to clinical risk, including hydration guidance, activity modification, medication considerations, symptom monitoring, and awareness of local cooling resources |
| Timing of delivery | Outreach initiated within 1-2 days of forecasted extreme heat event identification |
| Frequency / dose | Outreach attempted twice until successful per identified heat event, with prioritization based on clinical judgment |
| Tailoring | Educational content adapted based on members’ clinical conditions, medication profiles, and anticipated heat exposure |
| Operational supports | Centralized SharePoint hub containing standardized outreach scripts, decision-support prompts, workflow reminders, local cooling centers, and documentation guidance |
| Documentation standards | Structured contact logs and standardized fields within a dedicated care management technology platform to document outreach attempts, engagement status, and intervention delivery |

Footnote: Successful implementation of a climate-responsive care model required a robust foundation across benefit design, technology, and clinical operations. Key enablers included integrated data ecosystems; advanced analytics to identify, risk-stratify, and measure impact; and interoperable platforms supporting near real-time outreach and documentation. Automation and operational tools enabled scalability, while expanded scope of practice supported delivery within existing care management workflows. Transdisciplinary expertise spanning behavioral economics, human-centered design, environmental resiliency, and analytics ensured the microinterventions were evidence-based and member-centric.

**eTable 2. Implementation Outcomes of a Heat-Responsive Care Management Quality Improvement Initiative**

| **Implementation Domain** | **Outcome** | **Operational Definition** | **Observed Result** |
| --- | --- | --- | --- |
| Reach (Penetration) | Eligible members identified | Members enrolled in care management residing in census tracts with ≥2d consecutive days of forecasted extreme heat (WBGT >87° F) | 29% (3,978 of 13,712 eligible)  Standard: 1,915  Enhanced: 2,063 |
| Fidelity | Standard outreach rate | % of eligible members with ≥1 contact attempt for standard care management consistent with training and documentation requirements | Standard: 88.7% (1,698 of 1,915)  Enhanced: 88.0% (1,816 of 2,063) |
| Engagement | Standard engagement rate | % of members outreached for standard care management with successful two-way interaction with a nurse care manager | Standard: 86.5% (1,469 of 1,698)  Enhanced: 86.7% (1,574 of 1,816) |
| Adoption | Care manager participation | % assigned care managers trained and active | 100% (total N proprietary) |
|  | Care manager training | % care managers completing optional training | 67% (total N proprietary) |
| Feasibility | Weather data integration | Integration of third-party forecast data via API to enable automated member identification and outreach task generation | Achieved |
|  | Trigger logic fidelity | Proportion of member identification lists generated only when forecasted WBGT exceeded prespecified threshold of >87° F | 100% |
|  | Forecasted exposure (expected WBGT) | Forecasted WBGT during identified heat events exceeding prespecified threshold of >87° F | Mean forecasted WBGT: 89.3° F (range: 87.0 – 100.3° F) |
|  | Exposure confirmation (observed WBGT) | Observed WBGT during identified heat events exceeding prespecified threshold of >87° F | Mean observed WBGT: 91.6° F (range: 87.0 – 100.6° F) |
|  | Clinical eligibility accuracy | % of identified members meeting prespecified clinical eligibility criteria | 100% |
|  | Operational cadence | Successful generation of prespecified weekly member identification lists during the intervention period | 9 of 9 qualifying weekly lists successfully generated |
| **Implementation Domain** | **Outcome** | **Operational Definition** | **Observed Result** |
|  | Workflow activation | Creation of outreach tasks in care management platform | Achieved |
|  | Timeliness of activation | Outreach initiated within 1-2 days of identification | Achieved |
| Scalability | Volume supported | Total number of members successfully contacted during pilot period | N=3,380 |

Abbreviations: WBGT, wet bulb globe temperature

Footnote: Implementation outcomes were reported to characterize real-world feasibility and delivery within routine care management workflows. Observed rates were intended to inform operational expectations and not performance thresholds.
